# Improving Turn Movement Count Using Cooperative Feedback

**DOI:** 10.3390/s23249772

**Published:** 2023-12-12

**Authors:** Patrick Heyer-Wollenberg, Chengjin Lyu, Ljubomir Jovanov, Bart Goossens, Wilfried Philips

**Affiliations:** TELIN-IPI, Ghent University–imec, St-Pietersnieuwstraat 41, B-9000 Ghent, Belgium; chengjin.lyu@ugent.be (C.L.); ljubomir.jovanov@ugent.be (L.J.); bart.goossens@ugent.be (B.G.); wilfried.philips@ugent.be (W.P.)

**Keywords:** Turn Movement Count (TMC), cooperative vision, vehicle count, smart intersection, traffic analysis

## Abstract

In this paper, we propose a new cooperative method that improves the accuracy of Turn Movement Count (TMC) under challenging conditions by introducing contextual observations from the surrounding areas. The proposed method focuses on the correct identification of the movements in conditions where current methods have difficulties. Existing vision-based TMC systems are limited under heavy traffic conditions. The main problems for most existing methods are occlusions between vehicles that prevent the correct detection and tracking of the vehicles through the entire intersection and the assessment of the vehicle’s entry and exit points, incorrectly assigning the movement. The proposed method intends to overcome this incapability by sharing information with other observation systems located at neighboring intersections. Shared information is used in a cooperative scheme to infer the missing data, thereby improving the assessment that would otherwise not be counted or miscounted. Experimental evaluation of the system shows a clear improvement over related reference methods.

## 1. Introduction

*Turn Movement Count* (TMC) is the task of counting how many vehicles perform each of the possible movements at an intersection in a specific time period. It has been widely used in the applications of infrastructure planning, smart cities, and traffic optimization. Existing automated traffic analysis systems often underperform compared to human annotators, but they are able to annotate much larger datasets for extended periods of time. Despite these systems’ successes, they tend to miscount turn movements in situations with many simultaneously visible vehicles. These vehicles occlude each other, making it impossible to correctly identify the origin or destination of the vehicle. If a vehicle is occluded for most of its trajectory, current methods that rely on a single source of information cannot overcome this problem.

More recently, systems that implement multiple cameras at a single intersection have been introduced, with the goal of overcoming some of these problems in cases when a vehicle can be seen by at least one camera at all times. However, to ensure that all vehicles are in view, greater numbers of cameras are required and the computational cost is higher due to the larger amount of data to be processed. Although increasing the number of cameras observing a single intersection is not a cost-effective solution, traffic surveillance systems that observe the entry and exit streets at most intersections are already deployed in most cities. Other methods from the literature rely on information provided directly from vehicles. The available information varies depending on the vehicle types transiting through the area and the implemented technology. Information sources range from RFID tags that simply inform the system that a certain vehicle is present, to highly sophisticated systems common on autonomous vehicles. These latter systems obtain information on traffic environments from onboard sensors such as cameras, radars, and LIDAR.

In this work, we propose a system that utilizes existing camera infrastructure to perform TMC cooperatively. This system consists of multiple independent observational systems that collect and process local data at different locations *in parallel* and share relevant information between nearby intersections. The shared data provides a broader context to the observations at any given intersection, either by confirming previous motion estimations or by providing information about the vehicle’s movements before entering and after leaving the intersection. We postulate that with this additional information, a cooperative system should be capable of correctly evaluating a vehicle’s movement under conditions in which current systems would fail, given the same sensor configuration.

## 2. Related Work

Over the years, various innovative approaches have been proposed to solve the turn movement count problem using video analysis. Most of the methods described below employ a variation in the Detect-Track-Count paradigm, which consists of a set of *sequential* processes.

### 2.1. Detection

The Detection step consists of determining the regions of interest (ROI) in the image containing vehicles. Early methods used a variation in background subtraction to determine the ROIs, such as those presented in [1,2], by removing parts of the image that have not changed significantly over a certain number of frames. As these methods require multiple steps to be performed in sequence, they are often slower and more computationally expensive than more recent approaches.

More recent approaches rely on deep learning techniques to detect vehicles in the scene, such as in the works of [3,4,5]. These techniques have demonstrated better performance in detecting vehicles. In addition, these approaches are able to process algorithms such as YOLO [6] faster, a technique that provides accurate regions of interest from a single pass over the image. While improvements in detection speed and accuracy have contributed to a better TMC performance, they do not address high occlusion scenarios.

### 2.2. Tracking

The Tracking step of the process obtains the trajectories of previously detected vehicles as they move across the intersection. The most naive tracking methods, such as those presented by [7,8], extract the distances between the current and previously detected object positions. The trajectory is therefore described as a series of points in a sequence of images in which the vehicle has been detected. As these systems are frame-to-frame based, they require a high frame rate to track vehicles, given that the association of trajectory to a specific vehicle becomes inaccurate at low frame rates.

More sophisticated methods rely on a combination of visual features’ re-identification (described further below in Section 2.4) and the current position, to associate a trajectory to a vehicle. Such methods can correctly determine the location of an identified vehicle in a new frame, instead of relying on the proximity of detected positions to associate the tracks. Therefore, they allow for more robust trajectory assignment. This can be seen in the works of Liang et al. and Wojke et al. [9,10]. This approach reduces the uncertainty of the tracker while allowing for lower frame rates to be used, at the expense of higher computational complexity.

Other tracking systems, such as the one presented by Li et al. [11], essentially merge detection and tracking into a single process. This tracker passes a prediction of probable ROIs based on the current trajectory, providing the detector algorithm with a distribution of probable ROIs on which to perform detection. While this solution is the most complex of those discussed here, it is also the most reliable for continuous tracking, once a vehicle has been detected and tracking has started.

### 2.3. Movement Assignment and Counting

Movement assignment and counting is the last step taken via existing TMC methods. Based on the trajectory obtained from the tracking system, a turn movement is selected and counted based on a preexisting list of possible moves. Two main approaches exist for selecting the turn movement.

The first method consists of determining the entry and exit points of the vehicle by performing an intersection test between the vehicle’s trajectory and a predefined region of the intersection; the entry point is defined as the first region where the vehicle was detected, and the exit point corresponds to the last tracked position. This type of TMC can be seen in works such as [1,12,13,14]. When the detection algorithm is slow, this type of TMC becomes unreliable—the entry point will be incorrectly identified, and in cases where the vehicle is occluded while in the exit region, missed assignments will occur.

The second method consists of comparing the entire trajectory with previously known trajectories from the annotated data. These methods, as presented in [15,16,17], tend to be computationally expensive, since comparing whole trajectories is more complex than performing point-region intersection tests. The biggest issue in trajectory comparison is the precision of the tracker and the accuracy of the reference trajectory, since minor tracking deviations can generate confusion during evaluation.

### 2.4. Re-Identification

Re-identification consists of determining if an object of interest is detected—in this case a vehicle—and if it is the same as a previously detected vehicle. When an object has been detected, a series of identifying features are collected and compared to all previously collected feature sets. If the similarities between the features surpass a certain threshold, the object is considered the same as the one generating the initial feature set. While re-identification is not an essential part of TMC, some methods use it to increase the precision of assigning a trajectory to a vehicle. The main limitation of current vehicle re-identification methods is that the similarities of different vehicles tend to surpass the threshold for positive ID. That is, the feature set used to describe a previously observed vehicle may be similar enough to that of a different vehicle and cause incorrect identification, confusing the second vehicle for the first one.

This problem can be partially solved by increasing the similarity threshold required to match a newly detected vehicle to an existing ID. However, this comes with the main drawback of decreasing the number of vehicles correctly identified.

### 2.5. Other Data Sources

Although in this work we focus on conducting TMC using camera-based systems, other methods perform traffic analysis using data collected from different sources. These methods include systems based on radar, such as in [18,19], while others use 3D data captured via LIDAR, such as [20,21]. In recent years, a new concept has emerged to combine existing roadside sensors, such as those already described, with vehicle-mounted sensors such as LIDAR. Since autonomous driving vehicles already include such technologies, as described in [22], this integration would deliver more relevant information to the traffic system without increasing the cost of the infrastructure. The inclusion of these additional traffic data sources presents a great opportunity for research. However, a main limitation barring a wider application of these concepts is the limited types of data used in existing public infrastructure, where these kinds of systems are to be deployed.

## 3. Methodology

**Figure 1:**
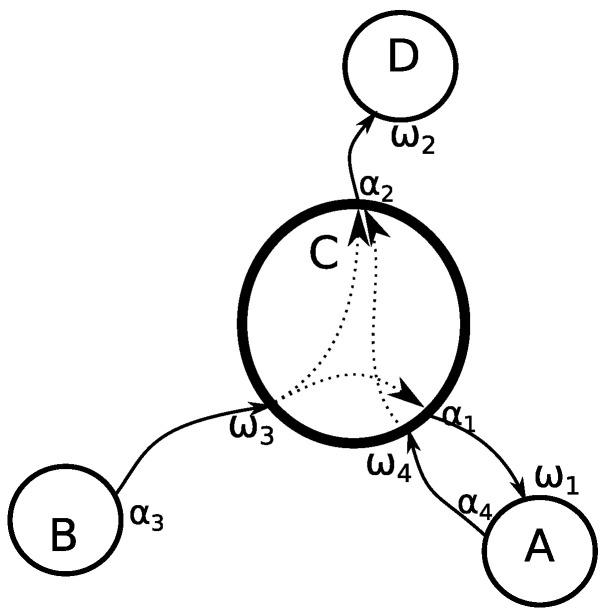
Visual representation of an intersection as a node C in a connected graph, with neighboring intersections *A*, *B*, *D*. Intersection *C* is connected with entry points $w_{3},w_{4}$, and exit points $a_{1},a_{2}$. Dotted lines represent possible movements through the node, representing the legal turn movements at that intersection.

We propose a cooperative feedback approach to address the vehicle occlusion problem affecting traffic analysis systems. Our approach gathers information from multiple points in surrounding areas. This information is used to assign a turn movement to a vehicle in cases where the assignment could not be made using information obtained only locally. We propose this approach based on the fact that the movement of a vehicle through an intersection is not an isolated event limited to the intersection, but rather part of a complex series of movements through an environment of interconnected roads, observed via traffic monitoring systems at multiple intersections. We represent the interconnections of roads as a directed multigraph, where each intersection is considered a node and each of the lanes connecting these intersections is an edge, as shown in Figure 1 where the main intersection *C* is connected to *A*, *B*, *D* using directional edges representing the traffic flow. We also consider all possible combinations of incoming and outgoing edges as the list of possible turn movements at that node, without considering the legality of such moves.

To determine how the information provided by the other intersections influences a TMC system, we propose a method formally described in Section 3.1. In Section 3.2, we describe the software implemented to test this system.

### 3.1. Formalization

Given the previously described assumptions, the problem can be formalized as follows:

- $U=\{u_{v}|v=1,\ldots ,V\}$, a set of objects of interest $u_{v}$ (vehicles in this case) where *V* denotes the cardinality of the set.
- $\Omega =\{\omega_{j}|j=1,\ldots ,J\}$, a set of incoming edges’ ends $\omega_{j}$ (entry points to intersections in this case), where *J* denotes the cardinality of the set.
- $A=\{\alpha_{k}|k=1,\ldots ,K\}$, a set of outgoing edges’ ends $\alpha_{k}$ (exit points to intersections in this case) where *K* denotes the cardinality of the set.
- $\Gamma =\{\gamma_{i}|\gamma_{i}\in \Omega ⋃A\}$, the set of edges’ ends $\gamma_{i}$ (accesses, whether entries or exits, to intersections in this case).
- A crossing $P(c_{l}):\Omega \to A$ is a function linking entries to exits according to the rule of association $P\left(c_{l}\right)=\left\{\left(\omega_{j},\alpha_{k}\right)|\omega_{j}\in \Omega ,\alpha_{k}\in A\right\}$, where a set of ordered pairs between entries $\omega_{j}$ and $\alpha_{k}$ exits on a crossing. Note that this allows us to model most types of intersections. We enforce the constraint that any given access $\gamma_{i}$ can only be part of, at most, one crossing $p(c_{l})$, noted $\gamma_{i}^{l}$, but within that intersection, it may participate in several ordered pairs, i.e., we assume free ends. We refer to an ordered pair $\left(\omega_{j}^{l},\alpha_{k}^{l}\right)$ as a *pathlink* $p_{jk}^{l}$; hence, a crossing is a set of intranodal pathlinks.
- $C=\{P\left(c_{l}\right)|l=1,\ldots ,L\}$, the set of intersections as described by its pathlinks $P(c_{l})$, with *L* as the cardinality of the set.
- A road network can be represented by a directed multigraph G=(C,E) of intersections and E=C×C and streets. Now, the incoming edges to a node form Ω, and the outgoing edges are in *A*. In other words, an edge is an ordered pair $e=(\alpha_{k},\omega_{j})$, with Γ as all the edges’ endpoints. Not all intersections will be monitored, but this is circumstantial. Furthermore, it is not critical to know the whole network G, and it suffices to know *C*.
- $S=\{s_{r}^{i}|r=1,\ldots ,R,i:\gamma_{i}\in \Gamma \}$, a set of observations obtained from the video analysis (acquired with some corresponding camera) $s_{r}^{i}$ looking at some intersection access $\gamma_{i}$, with *R* denoting the cardinality of the set. If a camera can monitor more than one access in one or more intersections, this can be represented by as many $s_{r}^{i}$ instances as required. If any camera does not monitor some access, there will not be a data source for that access. An access $\gamma_{i}$ may be observed via none, one, or more cameras. These observations will be noted as $s_{r}^{i}\left[t_{n}\right]$ with n=1,…,T set of timestamps.

At any given time $t_{n}$ of {T}, a vehicle $u_{v}$ may be detected using an observation algorithm $s_{r}^{i}$. A function $f_{detect}$ in Equation (1):

(1) $$\begin{array}{cc} f_{detect}: & \left\{T\}\times S\times U\to Bool=\{True,False,NA\right\}\\ \; & b=f_{detect}\left(t_{n},s_{r}^{i},u_{v}\right) \end{array}$$

indicates when the vehicle $u_{v}$ is being detected via some observer $s_{r}^{i}$ at the time $t_{n}$, $s_{r}^{i}\left[t_{n}\right]$. If the status of the detection *b* is True, this indicates that the vehicle is being detected. The outcome False indicates that the object is not being detected, while NA indicates that the detection cannot be confirmed (e.g., due to occlusions). Naturally, because $s_{r}^{i}$ monitors access $\gamma_{i}$ and given the constraint that one access can only be part of, at most, one intersection $p(c_{l})$, if $f_{detect}\left(t_{n},s_{r}^{i},u_{v}\right)=True$, we also know that object $u_{v}$ was at node $p(c_{l})$ at time $t_{n}$.

The problem can be stated as follows: Given a time *t*, an object $u_{v}$, an entry access $\omega_{j}$ at intersection $p(c_{l})$, noted $\omega_{j}^{l}$, and knowledge of the situation at the intersection *C* and observation *S*, *determine* the (most likely) exit access $\alpha_{k}$ in the same crossing $p(c_{l})$, noted $\alpha_{k}^{l}$ t with *g* as the node traversing function, which is unknown.

(2) $$\underset{\alpha_{k}^{l}}{\mathrm{argmax}}\;P_{r}\left(g\left(t_{n},u_{v},\omega_{j};C,S\right)=\alpha_{k}\right)$$

In other words, determine the most likely outgoing end of the pathlink $\left(\omega_{j}^{l},\alpha_{k}^{l}\right)$, followed by the vehicle $u_{v}$ traveling road network G at time $t_{n}$ exploiting the info in *S*. This is solved using the Nelder–Mead algorithm [23] by iteratively adjusting the shape of a graph to find the lowest or highest point, depending on the goal. Shape optimization continues until passing a convergence threshold or reaching a stopping condition.

### 3.2. The Algorithm

**Figure 2:**
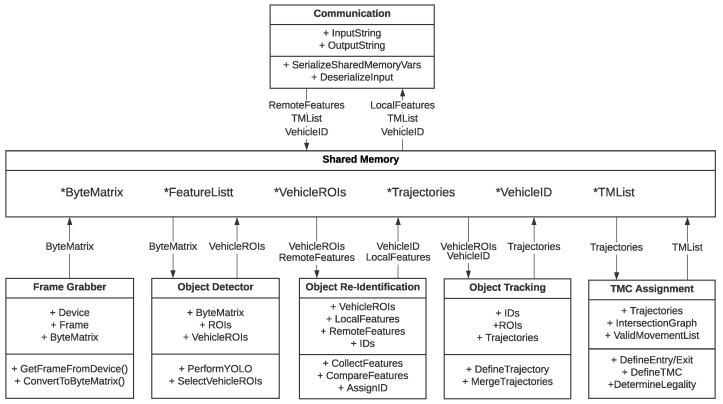
Illustrative data exchange among different modules of the architecture proposed. All modules are executed in parallel and data exchange is bidirectional and asynchronous.

The proposed solution is organized as a modular platform that executes different algorithms in parallel. Each algorithm is implemented as an independent plugin, sharing information using a shared memory whiteboard model. An illustration of the proposed algorithm is shown in Figure 2.

#### 3.2.1. Data Acquisition

This module obtains the most recent unprocessed video frame from the device and associates a timestamp to it, then saves the frame to memory and shares it with the other plugins using the shared whiteboard. The protocols supported by this plugin are *RTSP* [24], *TrafiSense2 Dual* thermal camera, *iDS uEye* camera, and local video files or image sequences.

#### 3.2.2. Detection

**Figure 3:**
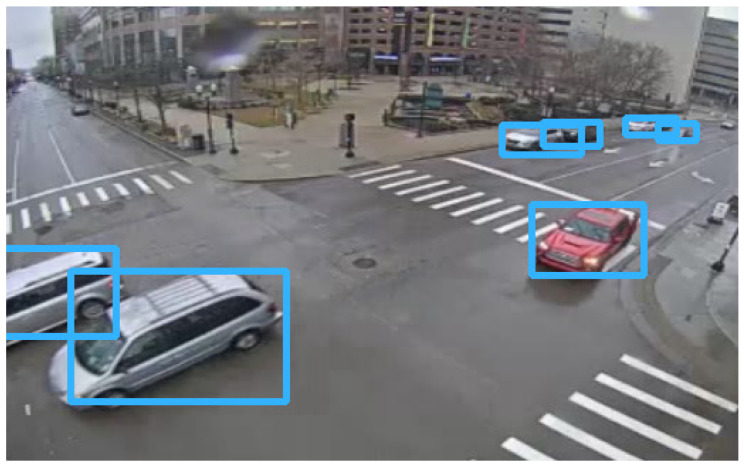
Visual representation of resulting bounding boxes defined by the detection algorithm.

This plugin relies on the YOLO version 4 implementation provided by OpenCV, [25] using the parameters shown in Table 1 trained on the COCO dataset [26] and considering only the traffic-related objects from its multi-class output. The algorithm determines the position and bounding boxes of the vehicles in the scene, as illustrated in Figure 3. Once the bounding box is determined, it is cropped and shared along with its view space coordinates.

#### 3.2.3. Feature Collection and Re-Identification

**Figure 4:**
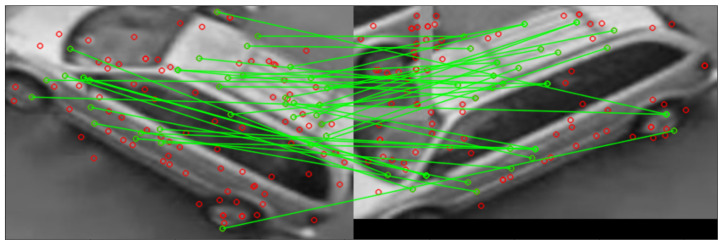
Feature matching during the re-identification process. *Red circles* features detected via the ORB algorithm. *Green lines* matching features.

This plugin extracts recognizable features from the bounding box using the ORB algorithm [27]. A sample of the feature match progress is shown in Figure 4. A set of newly extracted features are compared to either the previously known features collected via this processing node, or to those received from other locations via the network, using the *Hamming distance* defined in Equation (3) where the features *a* from the current camera image are compared to features *b* stored from previously collected features, as well as where $a_{i}$ and $b_{i}$ are the individual features.

(3) $$d_{hamming}\left(a,b\right)=\sum_{i=0}^{n-1}\left(a_{i}⊕b_{i}\right)$$

If there is a match, the vehicle is considered re-identified and the known ID is assigned. If the comparison does not pass the threshold, the vehicle is regarded as *unknown* and a new ID is assigned. Once the ID is assigned, the system shares it, appending its features to the existing feature set.

#### 3.2.4. Tracking

**Figure 5:**
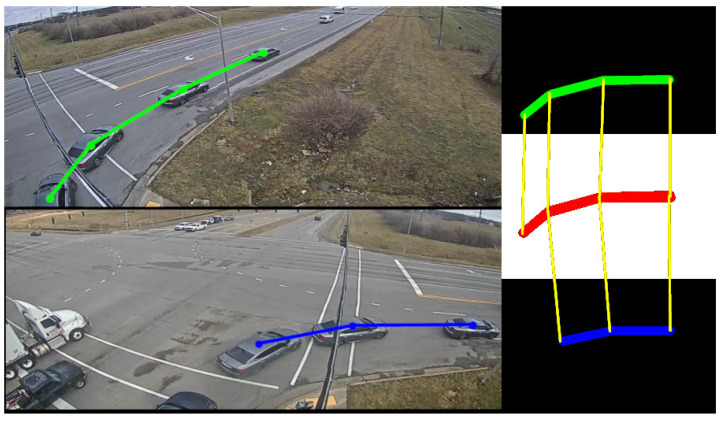
Tracking and smoothing process. Image-space trajectories projected to top-view *blue and green lines* and combined to obtain the final trajectory in top-view space *red line*.

This module follows the vehicle through the scene. The position obtained via the detector is projected to a top-view representation. In addition, the position of each detected object is tracked using a Kalman tracker in combination with the ID provided by the re-identification algorithm. Once the top-view trajectory has been determined for all cameras, the trajectories are merged based on their associated ID, using the Frechet distance as shown in Algorithm 1 and in Figure 5. This algorithm measures the similarity between two curves that maintain a certain proximity by recursively calculating the Euclidean distance of the points that belong to the curves. If the resulting distance is below a certain threshold, the trajectories are merged using point averaging. Thus, the resulting trajectory is stored as an observation *S* for the intersection *C*. The stored trajectories are then used by the turn movement assignment algorithm to select both the most probable route taken and the appropriate TMC.

#### 3.2.5. Turn Movement Assignment

**Figure 6:**
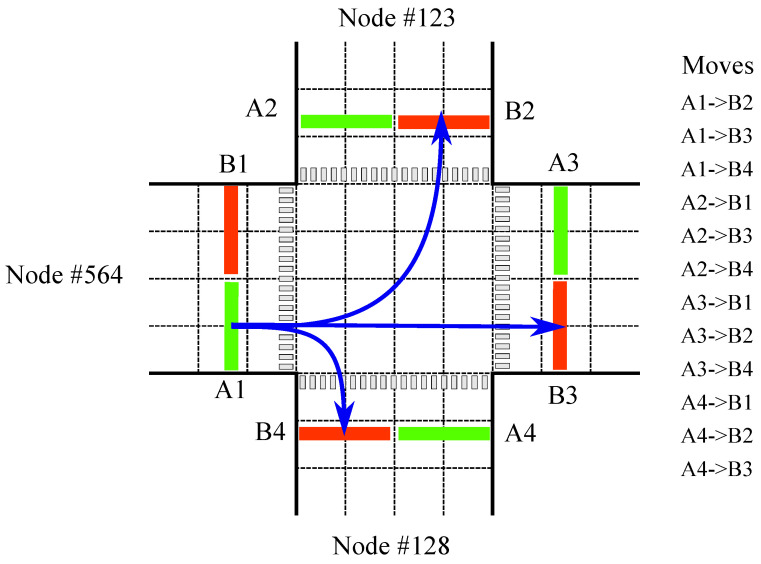
Visual representation of the proposed scene model at one intersection. Blue arrows indicate three legal turn movements at this specific intersection starting from A1: left (A1→B2), straight (A1→B3), and right (A1→B4).

The system determines the most probable trajectory $\left(\omega_{j}^{l},\alpha_{k}^{l}\right)$ for a vehicle $u_{v}$ based on its entry point $\omega_{j}$ and the observations collected via the tracking module using Equation (2). This is performed by comparing the observed trajectory, aligning the sequences in a non-linear manner to a series of predefined turn movements, as shown in Figure 6, and finding the optimal match by stretching or compressing one of the trajectories to match the other, as described in Algorithm 2. The trajectory that requires the smallest change is considered the best match to the known legal turn movement.
**Algorithm 1** MergeTrajectories**function** MergeTrajectories (splines,threshold)      trajectory←∅      **if** splines is not empty **then**            **for** i←0 to length(splines)−1 **do**                  **for** j←i+1 to length(splines) **do**                        distance←CalculateEuclideanDistance(splines[i],splines[j])                        **if** distance<threshold **then**                               mergedSpline←AveragePoints(splines[i],splines[j])                               trajectory←trajectory∪{mergedSpline}                         **end if**                   **end for**             **end for**       **end if**       **return** trajectory **end function**

**Algorithm 2** TrajectorySimilarityEvaluation
  1:**function** TSS(A,B): float  2:      n←lengthofA  3:      m←lengthofB  4:      DP←a2Darrayofsize(n+1)×(m+1)  5:      **for** i←1 **to** *n* **do**  6:            **for** j←1 **to** *m* **do**  7:                  cost←distancebetweenA[i]andB[j]  8:                  DP[i][j]←cost+min(DP[i−1][j],DP[i][j−1],DP[i−1][j−1])  9:            **end for**10:      **end for**11:      **return** DP[n][m]12:
**end function**



Once the most probable trajectory is assigned, it is stored along with the vehicle ID and used for future comparisons with the trajectories obtained by human observers. In addition, the computed TMC is communicated to other nodes on the road network G to be used in further computations.

#### 3.2.6. Communication

This module collects all available information shared by the other modules of its node and sends it to other nodes on the network. It also receives incoming data from other nodes and integrates it into the node’s shared whiteboard. As the main component of the cooperative system, this module allows the node access to information about the environment that the locally connected sensors cannot collect. The information exchange uses a low-latency broadcasting messaging protocol that sends messages to all the other nodes simultaneously. In addition, the communication system also sends messages at each *vehicle entry event*, when a vehicle has been re-identified via a new node, and *vehicle exit event*, when the vehicle has left an intersection. The data communicated is user-defined; to reduce the amount of data exchange in our experiments, data sharing was limited to: *originating node, timestamp, re-identification ORB features, vehicle IDs, and entry/exit point*.

In addition, this module continuously reviews the events provided by the other systems and compares the entry/exit events to determine if the vehicle has been re-identified via another node. If re-identification occurs, the turn movement assignment is confirmed, thereby giving a higher certainty of a correct assessment.

## 4. Experiments

To evaluate the influence the proposed cooperation scheme has on the overall performance of a TMC system, we conducted a comparison between four scenarios, using the same dataset. The scenarios were a no-cooperation setup, a partial TMC confirmation, a complete TMC confirmation, and a partial blackout setup. We assessed the accuracy of TMC assignments compared to the ground truth manual annotations. We hypothesized that the TMC system would have the highest performance when maximum information was shared between the different nodes.

### 4.1. Experimental Setup

**Figure 7:**
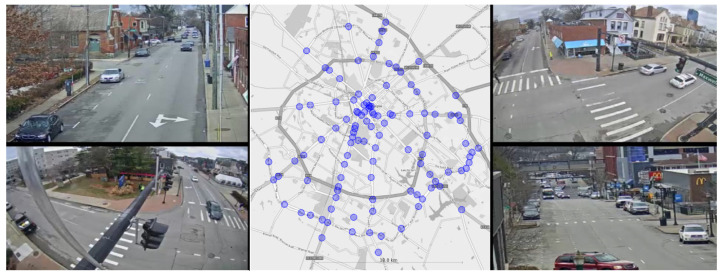
Illustrative frames of video captured at one of the intersections and approximated locations of all the cameras recorded.

To correctly assess how the knowledge of its surroundings affects the result of a TMC algorithm, the dataset used should provide the context of surrounding intersections. To the best of our knowledge, no such dataset was available. A new dataset was captured using publicly accessible traffic cameras provided by the Lexington-Fayette Urban County Government in Lexington, KY, USA. The dataset was recorded from a total of 90 intersections, each equipped with four cameras pointing in the general direction of the legs of the intersection. Recordings were 1280 × 720 pixels, collected for 30 min at 25 frames per second. One issue we had to address is that the cameras in this system were destined for human traffic monitoring. Therefore, these cameras have not been calibrated or configured for software analysis. Sample images captured at one of the intersections used in this dataset can be seen in Figure 7. To overcome the problem of calibrations, the views were manually projected to align with the observable ground plane.

Since the full context was not available for all intersections, a subset was selected. We chose intersections whose adjacent intersections were also recorded. The videos from these intersections were manually labeled by assigning an identifier to each leg of the intersection, enumerating each possible movement to be performed, and finally, assigning one of the possible movements to each vehicle in the scene. The data from the adjacent intersections were labeled and assigned the same unique ID to each vehicle in all scenes. As the experiment did not allow for on-site analysis, the experiments were performed offline on a single computer. To best replicate real-world conditions where each intersection would be processed via a single *edge* device such as an Intel NUC or a similar low-power unit, each node was executed on a single thread of the host unit, limiting the RAM available to 1 GB and with no GPU acceleration. Meanwhile, the network connection was set to 10 Mbps with an average simulated latency of 100–150 ms, which is similar to the latency found on low-cost public infrastructure solutions. For each experiment, the system simulated five processing nodes simultaneously, i.e., one main central node and four secondary nodes. The results reported in Section 4.3 only consider the results of the central node. The secondary nodes are used to provide context to the main node to increase its accuracy, but are not used directly for the evaluation. In this way, the systems are compared under the most similar conditions possible.

### 4.2. Experimental Scenarios

**Figure 8:**
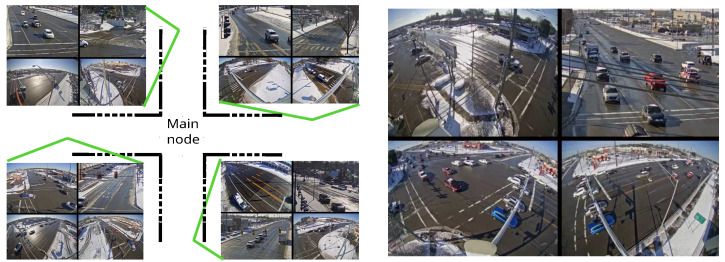
Illustrative distribution of a common four-legged intersection with one central node and four secondary nodes that communicate.

These scenarios were designed to test our hypothesis that “A cooperative system will surpass a non-cooperative system under the same conditions”. Specifically, we selected the three most circulated intersections, each in a traditional crossroad configuration (i.e., four-legged symmetric intersection). For each intersection, the central intersection area acted as the main point of interest, and the four surrounding nodes served as cooperative inputs. An illustration of a typical four-legged intersection can be seen in Figure 8. The four scenarios are described next.

**Scenario 1: No cooperation.** Cooperation is entirely disabled. Information provided by other nodes is *not* used to determine the vehicle’s movement. To maintain a similar workload of the system, we left communication enabled, but configured the main node to ignore any information provided by the other nodes of the network. This reflects the basic scenario of how most TMC systems are tested. That is, a standalone system that is only capable of using locally collected data. This also reflects the fundamental behavior of non-cooperative systems typically found in the literature. Therefore, we consider this scenario as a baseline for the comparison of a cooperative system, as all other conditions remain equal.

**Scenario 2: Partial TMC confirmation.** In this scenario, the data from other nodes is only used if the entry/exit points cannot be determined using local data. This scenario represents the main cases where the vehicle is occluded during the initial or final parts of its trajectory. This can be reduced to the following steps:

(1) If entry and exit points are defined:
  - Store trajectory and exit.
(2) If no entry point is defined:
  - Request re-identification.
  - If the re-identification is positive:
    - Assign the vehicle entry point based on the location where the vehicle was initially identified.
  - If the re-identification is negative:
    - Assign the vehicle entry point based on the closest available point.
(3) If no exit point is defined:
  - Await a positive re-identification reported by another node.
  - If the re-identification is positive:
    - Assign the exit point to the leg of the intersection connected to that node.
  - If the re-identification is negative:
    - Assign the vehicle exit point based on the closest available point.

We limit the cooperation in this scenario to undetermined cases. Therefore, the system will not perform any correction to incorrectly assessed movements. Consequently, incorrect assignments made on the central node, not related to an incomplete trajectory, will remain incorrect even if other nodes perform correct re-identification.

**Scenario 3: Complete TMC confirmation.** In this scenario, we take full advantage of the cooperative mode of the system. Each node performs the turn movement assignment using the local data, while also confirming with other nodes following the steps listed below:

(1) Each node performs the turn movement assignment using the local data.
(2) Confirm the entry point by:
  - Performing a re-identification of the vehicle.
  - Determining if the assigned entry point coincides with the previously identified node.
(3) Confirm the exit point by:
  - Awaiting a positive re-identification reported by another node.
  - Determining if the assigned exit point coincides with the subsequently identified node.
(4) If more than one node presents an entry/exit:
  - Maintain the locally obtained trajectory and exit as probable misidentification occurred.
(5) In case of any discrepancy between local and remote ID:
  - Re-assign the points if the re-identification confidence level is above the threshold.
  - Maintain original points if the re-identification confidence level is below the threshold (assumed misidentification).

In addition, the node also adds vehicle IDs with a turn movement when two neighboring nodes indicate the route to the intersection as the exit point and entry point, respectively. Therefore, the node manages to assign probable turn movements even to occluded vehicles, based on the information provided by its peers.

**Figure 9:**
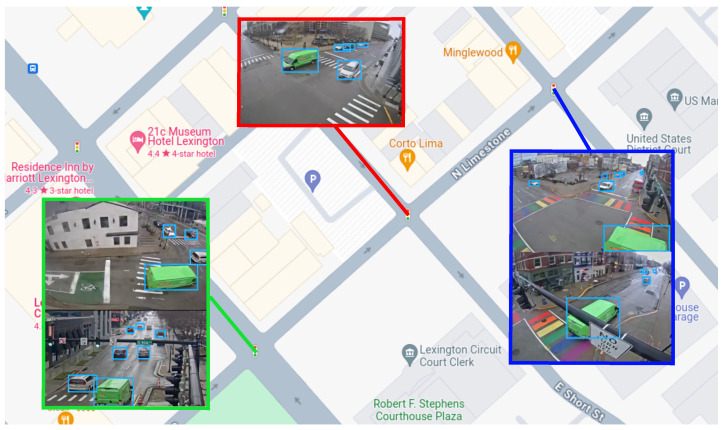
Illustrative example of the cooperation of the system. In this scenario, it is possible to infer the trajectory of the vehicle across the middle intersection (red) from the information provided by the neighboring nodes (green, blue), even if the camera at the central intersection was obstructed.

**Scenario 4: Partial blackout.** In this scenario, the system is configured with the same cooperative capabilities as in scenario 3—*Complete*. However, the locally obtained data is blocked in order to simulate a failure of the cameras. This is a common situation when traffic accidents damage the sensing infrastructure. Traffic behavior analysis becomes more important in these cases, as the conditions at the intersection produce unexpected traffic flows. In order to make its turn movement assignment with these limiting conditions, the node depends entirely on the cooperative aspect of the system and the data provided by the surrounding nodes. Under this scenario, the system should be able to correctly assess those cases where a vehicle is identified by two of the adjacent nodes and assign the turn movement based on this. While this scenario represents an extreme case where a system that only uses local information is completely incapable of performing correctly, it presents an opportunity to determine if cooperation is beneficial in cases of technical failures, such as the one illustrated in Figure 9.

The architecture is configured in such a way that the plugins of the platform can be activated based on the cooperative level being used in each tested scenario, which can be seen in Algorithm 3. This is used to dynamically change how each node of the network uses the context provided by the other nodes and what information is to be shared using the communication plugin.
**Algorithm 3** Simplified plugin call pseudocode  1:trajectory ← get_reduced_trajectory()  2:**if** cooperationlevel=1 **then**  3:      **if** notEntryPoint **then**  4:            **Assign closest entry point to trajectory**  5:      **end if**  6:      **if** notExitPoint **then**  7:            **Assign closest exit point to trajectory**  8:      **end if**  9:**end if**10:**if** cooperationlevel=2 **then**11:      **if** notEntryPoint **then**12:            trajectory.Entry←ReIdentifyEntry(features)13:      **end if**14:      **if** notExitPoint **then**15:            trajectory.Exit←ReIdentifyExit(features)16:      **end if**17:**end if**18:**if** cooperationlevel=3 **then**19:      **if** ReIdentifyEntry(features) > *threshold* **then**20:            trajectory.Entry←NewEntry21:      **end if**22:      **if** ReIdentifyExit(features) > *threshould* **then**23:            trajectory.Exit←NewExit24:      **end if**25:**end if**26:TM ← TrajectorySimilarityEvaluation(trajectory)27:AssignTMC(*TM*)

### 4.3. Experimental Results

The system was applied to three intersections, with a total of 890 manually labeled turn movements.

**Figure 10:**
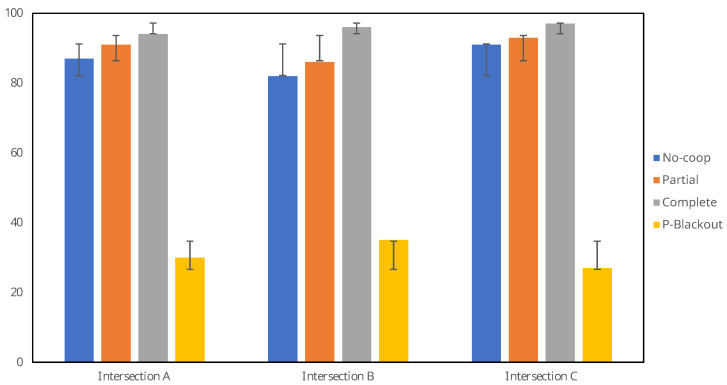
Correctly assigned turn movements in different scenarios.

As shown in Table 2 and in Figure 10, Scenario 3—*Complete*—achieved the highest average at 95.65% ±1.55 correctly identified movements and reached a performance of 97% on Intersection 3, outperforming the other scenarios by 4% to 70%. On scenario 4—*Partial Blackout*, the system was capable of correctly assigning on average 30.67% ±4.04 of the turn movements. Systems without a cooperative feature would be incapable of this task because the local data has been blocked, where such systems would identify zero vehicles.

Table 2 also shows that the standard deviation (STD) is smaller in more cooperative scenarios. This shows a clear tendency for an overall higher performance when the system shares more information.

These results can also be contrasted in Table 3, showing a clear increase in the F1 score when the system uses cooperative mode. The clear advantage of the use of cooperation in scenario 4 *P-Blackout* can also be seen, in contrast to a standard system, where the score without cooperation would be zero. It should also be noted that in scenarios 2 *Partial* and 3 *Complete*, cooperation reduces the false negative rate without inducing a large increase in the false positive rate, demonstrating that cooperation does not increase incorrect turn movement assignments.

(4) $$\mathrm{IDF}1=\frac{2\times \mathrm{TP}}{2\times \mathrm{TP}+\mathrm{FP}+\mathrm{FN}}$$

Further testing is required due to the relatively small dataset used in these experiments. Nevertheless, we note a tendency for performance improvements when using cooperative methods compared to non-comparative ones. Moreover, this system is capable of correctly inferring the information of an intersection without directly observing it.

## 5. Conclusions

While the TMC results of the proposed system, when functioning in non-cooperative mode, are similar to the published results in the literature, the integration of cooperation significantly improves the TMC performance. Additionally, it is noteworthy that by integrating a cooperative scheme into the algorithm, it is possible to perform TMC under adverse conditions where one part of the system is incapable of collecting data on its own. This system will be more robust and reliable in conditions where the correct assessment of movements is the most important factor when making decisions about infrastructure improvements.

The main contribution of a cooperative system, compared to non-cooperative systems, is that the proposed system is capable of determining the movements of vehicles even when no direct observation of the intersection is possible. As the cooperation of the system does not depend on any particular detection or tracking algorithm, the proposed system can be integrated with other existing TMC methods, providing additional certainty. The experiments described here demonstrate that the system is highly scalable, as each node is only required to both analyze the data provided by the cameras at one intersection and communicate with the directly neighboring intersection, e.g., 3–5 neighbors in most common urban scenarios. This scalability at a relatively low cost, compared with a centralized solution, allows for ad hoc expansion of the system. We expect to expand this research to evaluate the system in real-world scenarios using real *edge* hardware solutions. We also plan to integrate additional sensing capabilities such as thermal cameras, radar, and LIDAR, as these sensors provide additional information, expanding the possibilities of better infrastructure planning. We are also exploring the possibility of expanding the system to observe larger areas, such as highways and interstates, to perform long-term vehicle tracking. Once these sensing technologies have been integrated into the system, we plan to deploy the complete system in real-world scenarios to improve the local capabilities of traffic analysis.

## Figures and Tables

**Table 1:** Parameters used in the training of YOLO using the COCO dataset.

Parameter	Value	Parameter	Value	Parameter	Value
batch	64	momentum	0.9	learning_rate	0.001
subdivisions	16	decay	0.0005	burn_in	1000
width	608	saturation	1.5	max_batches	500,200
height	608	exposure	1.5	policy	steps
channels	3	hue	0.1	steps	400,000, 450,000

**Table 2:** Percentage of Correctly Assigned Turn Movements for the three intersections under the four scenarios.

	Intersection A	Intersection B	Intersection C	Average	STD
**No-Coop**	87%	82%	91%	86.67%	4.51
**Partial**	91%	86%	93%	90.00%	3.61
**Complete**	**94%**	**96%**	**97%**	**95.65%**	**1.55**
**P-Blackout**	30%	35%	27%	30.67%	4.04

**Table 3:** IDF1 score of the TMC under different scenarios on a total of 890 different turn movements. **IDF1** *score obtained using Equation* (4); **TP** *true positive*; **FP** *false positive*; **FN** *false negative*.

	IDF1	TP	FP	FN
No-Coop	0.7120	55.28	24.49	20.22
Partial	0.7464	59.55	21.01	19.43
Complete	**0.7799**	**63.93**	21.91	**14.15**
P-Blackout	0.4786	31.46	**9.77**	58.76

## Data Availability

The data and code can be requested by contacting the corresponding author.

## Abbreviations

The following abbreviations are used in this manuscript:

ID	Identification
LIDAR	Light Detection and Ranging
RFID	Radio Frequency Identification
ROI	Region Of Interest
RTSP	Real Time Streaming Protocol
STD	Standard Deviation
TMC	Turn Movement Count
YOLO	You Only Look Once
